# Ectopic microRNAs used to preserve human mesenchymal stem cell potency and epigenetics

**DOI:** 10.17179/excli2018-1274

**Published:** 2018-06-14

**Authors:** Navid Ghasemzadeh, Fatemeh Pourrajab, Ali Dehghani Firoozabadi, Seyedhossein Hekmatimoghaddam, Fatemeh Haghiralsadat

**Affiliations:** 1 Department of Biochemistry and Molecular Biology, School of Medicine, Shahid Sadoughi University of Medical Sciences, Yazd, Iran; 2 Yazd Cardiovascular Research Center, Shahid Sadoughi University of Medical Sciences, Yazd, Iran; 3 Hematology & Oncology Research Center, Shahid Sadoughi University of Medical Sciences, Yazd, Iran; 4 Department of Laboratory Sciences, School of Paramedicine, Shahid Sadoughi University of Medical Sciences, Yazd, Iran; 5 Department of Advanced Medical Sciences and Technologies, School of Paramedicine, Shahid Sadoughi University of Medical Sciences, Yazd, Iran

**Keywords:** epigenetic, human mesenchymal stem cells (hMSCs), miRNA, potency, regenerative medicine

## Abstract

Human mesenchymal stem cells (hMSCs) have remarkable potential for use in regenerative medicine. However, one of the great challenges is preserving their potency for long time. This study investigated the effect of miRNA ectopic expression on their proliferation and also on the expression level of Parp1 as an epigenetic switch preserving pluripotency in hMSCs. A cationic liposome was prepared as an efficient carrier for miRNA delivery. The miRNA loading efficiency and physical stability of vesicles were measured, and their scanning electron microscopic shapes determined. hMSCs were transfected with miR-302a and miR-34a followed by assessment of their proliferation potency with MTT assay and measurement of the expression of Parp1 by quantitative polymerase chain reaction (QPCR). Cell transfection with miR-302a and miR-34a efficiently and differentially affects the proliferation potency of hMSCs and the expression level of Parp1 as the key epigenetic factor involved in pluripotency. While miR-302a increases Parp1 expression, miR-34a suppresses it significantly, showing differential effects. Our results demonstrated that miRNA-based treatments represent efficient therapeutic systems and hold a great promise for future use in regenerative medicine through modification of hMSC pluripotency and epigenome.

## Introduction

Human mesenchymal stem cells (hMSCs) hold a great promise for future therapeutic use in regenerative medicine. They have peculiar capability in replacing damaged cells in infarcted organs, and have an intrinsic renewal capacity (Pourrajab et al., 2013[15], 2014[18])*.* As indicated by studies, hMSCs undergo senescence during their life, and gradually lose their proliferation and differentiation capacity (Wagner et al., 2008[21]; Gang et al., 2007[9])*. *Senescent hMSCs have a limited lifespan, and exhibit reduced differentiation potential upon prolonged *in vitro* culture. This phenomenon limits their therapeutic applications. Thus, analysis of *in vitro* treatments to inhibit senescence in hMSCs is crucial for basic research as well as for quality control in cellular therapy (Pourrajab et al., 2014[18]; Wagner et al., 2008[21]). Signaling pathways and growth factors that preserve the pluripotency of stem cells including hMSCs and their lineage-specific differentiation and gene expression have been a major focus of stem cell research (Gang et al., 2007[9]; Li et al., 2017;[13] Doege et al., 2012[6]). Among them, our attention is to a key epigenetic factor, namely poly(ADP-ribose) polymerase-1 (Parp1), known for an early role in establishment of the epigenetic marks necessary for promoting the expression of pluripotency factors. Parp1 activation is an initial and essential stage necessary for reprogramming of somatic cells into induced pluripotent stem cells (iPSCs) wherein the expression of pluripotency factors is required. Parp1 is also known as a safeguard of pluripotency in embryonic stem cells (ESCs) (Doege et al., 2012[6]; Roper et al., 2014[19]; Jiang et al., 2015[11]). Parp1 is localized in nucleus wherein it modulates some biological events such as DNA repair, DNA replication, DNA transcription and DNA methylation. Parp1 is now recognized as one of the most important determinants of stemness and pluripotency in stem cells (Doege et al., 2012[6]; Roper et al., 2014[19]; Son et al., 2013[20]). 

On the other side, several *in vivo*/*in vitro* studies report that miRNAs are key modulators influencing signaling pathways in stem cells and act as master switchers governing senescence programs. Therefore, miRNAs hold the great promise in switching off/on cellular senescence/reprogramming to rejuvenate stem cells toward regenerative process (Pourrajab et al., 2014[17], 2015[16])*. *In this regard, numerous studies have reported miR-302 as a pluripotency-specific marker which plays critical role in preserving cellular proliferation, maintaining pluripotency and lineage differentiation in human stem cells. Its expression is an early and essential stage of somatic cell reprogramming. In contrast, miR-34a has attracted a lot of attention as a tumor suppressor molecule which, through several pathways, induces cell cycle arrest, senescence and apoptosis in proliferating cells (Wang et al., 2013[22]; Agostini and Knight, 2014[1]; Lee et al., 2013[12]; Gao et al., 2015[10])*.*

This study aimed to test a state-of-the-art theory which states microRNA-based treatment can modulate cellular mechanisms in proliferation capacity and the expression of key genes involved in early stages of pluripotency. Understanding the microRNA signature interactions, in conjunction with cell signaling, is critical for development of improved strategies to reprogram differentiated cells or direct differentiation of pluripotent cells (Pourrajab et al., 2014[17], 2015[16]). The present study investigated differential effects of miR-302a and miR-34a on hMSCs proliferation and the expression of the key epigenetic factor Parp1 involved in preserving stem cell potency and self renewal capacity. The efficacy of miRNA-containing liposomes for hMSC transfection was compared with free miRNA delivery (Pakunlu et al., 2006[14]; Wu et al., 2013[23]; Ando et al., 2013[2]). 

## Materials and Methods

### Materials

The methoxy polyethylene glycol-conjugated 1,2-distearoyl-sn-glycero-3-phosphoethanolamine (DSPE-MPEG) and soybean phosphatidylcholine (SPC) were purchased from Lipoid GmbH (Germany); cholesterol, thiazolyl blue tetrazolium bromide (MTT), NAHCO_3_ and fluorescent dye FAM (6-carboxyfluorescein) from Sigma-Aldrich Co (USA); and 1,2-dioleoyloxy-3-(trimethylammonio) propane (DOTAP) from Avanti Polar Lipids (USA). Dulbecco's modified eagle medium (DMEM) low glucose, Glutamax® supplement, pyruvate, phosphate-buffered saline (PBS) tablets, penicillin/streptomycin/amphotericin B and trypsine-EDTA were purchased from Gibco (USA). Fetal bovine serum (FBS) was obtained from Invitrogen (USA). The human bone marrow-derived hMSC line S1939 was sourced from Royan Institute, Iran. The mature miRNA sequence was obtained from the miRBase database (http://www.mirbase.org). MicroRNA oligonucleotide miR-302a and miR-34a were prepared ready-to-use from Qiagen, Germany.

### Preparation of cationic liposomes and lipoplexes

The liposome vesicles were prepared using a 70:20:30 molar ratio of SPC: cholesterol: DOTAP in presence of 5 % DSPE-MPEG. First, the lipid phase was dissolved in chloroform and allowed drying to form a thin film. Then a hydration step was performed using ammonium sulfate at 65 °C and sonication for 30 min. Ammonium sulfate covering small unilamellar vesicles was finely replaced with phosphate buffered saline (PBS) by dialysis for 2 hrs at 25 °C. Liposomes were incubated with miRNA for 30 min to prepare liposome-miRNA complexes (lipoplexes or LP-miRNAs). The effect of PEGylation was evaluated with several characterization methods. The lipids/miRNA mass ratio was 1 mg/ 10-100 μg, according to the references and based on the results obtained previously from gel electrophoresis assays (Pakunlu et al., 2006[14]; Wu et al., 2013[23]; Ando et al., 2013[2])*.*

### Physico-chemical characterization of complexes

Particle size, superficial zeta potential, hydrodynamic diameter and the polydispersity index (PDI) were determined by dynamic light scattering (DLS) (on a Zeta PALS, Brookhaven Corp Instruments, USA), according to the manufacturer's instructions and at room temperature by dilution with deionized water. All measurements were carried out in four consecutive attempts. Each parameter was measured three times, and average values and standard deviations were recorded (Pakunlu et al., 2006[14]; Wu et al., 2013[23]; Ando et al., 2013[2])*.*

### Loading efficiency and morphology of liposomes and lipoplexes 

**Figure 1:**
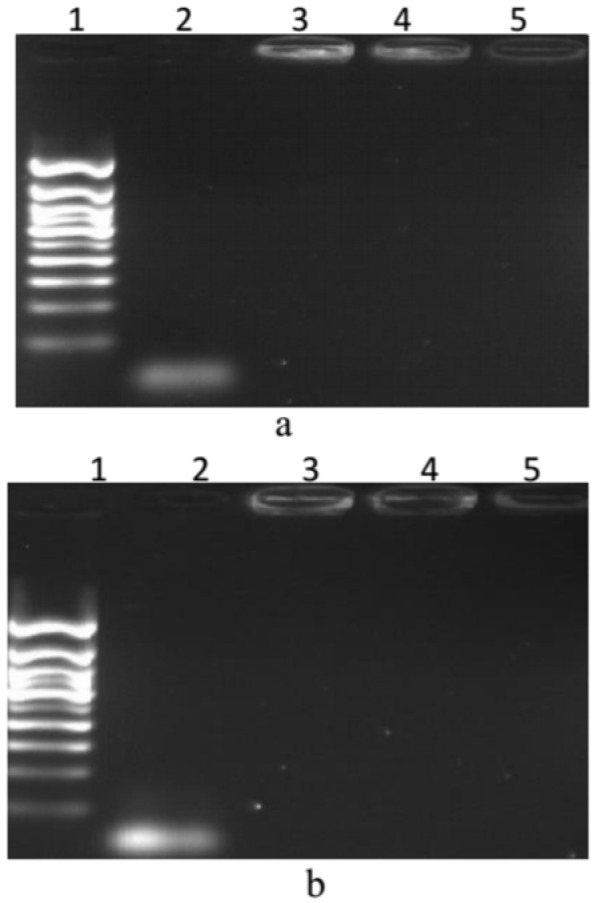
Agarose gel electrophoresis of free miRNA and miRNA loaded in liposome vesicles to determine the most effective ratio of miRNA (μg): liposome (mg). (a): lipoplexes with miR-302a; and (b): lipoplexes with miR-34a. Lane 1 = DNA ladder; lane 2 = free miRNA (3 µL); lane 3 = lipoplex (12.5 µL liposome, 0.9 µL miRNA); lane 4 = lipoplex (15 µL liposome, 0.9 µL miRNA); lane 5 = free liposome (4 µL).

For determination of loading efficiency of lipoplexes**,** various concentrations of naked miRNA (10-100 μg of miRNA) per 1 mg lipid (15-12.5 μL) were added to the provided liposomes, and then applied to ethidium bromide-containing agarose gel (2 %) electrophoresis (30 min electrophoresis at 80 V) to determine the miRNA loading by cationic liposome. Briefly, 4 μL of suspension was mixed with 1 μL of 4× DNA loading buffer (Biolabs, USA). Various concentration ratios of liposome to miRNA (12.5/0.9, and 15/0.9) in comparison with free liposome (4 μL) were analyzed to choose the maximum loading capability of miRNA in liposome (Pakunlu et al., 2006[14]; Wu et al., 2013[23]; Ando et al., 2013[2]). The DNA ladder (Biolabs, USA) was used as marker. The images were obtained after UV exposure in a gel-documentation system (UVP, Cambridge, UK) (Figure 1a and 1b(Fig. 1)). 

For morphology determination of lipoplexes, scanning electron microscopy (SEM, model KYKY EM3200, China) was used. Briefly, the prepared thin layers of samples were dried and coated with gold to make it electrically conductive using physical vapor deposition method by sputter coater instrument (model SBC 12, KYKY, China). The mixture of argon gas and gold ions with a positive charge were physically perched on the surface of negatively-charged sample. The SEM image was then provided at maximum voltage of 26 KV.

### Measuring quantity of loading and stability of the prepared lipoplexes

UV spectroscopy (PG Instruments, UK) at the wavelength of 260 nm was used to evaluate the amount of free (unloaded) miRNA. The prepared lipoplex solution was first centrifuged (35,000 rpm at 4 °C) to precipitate lipoplexes and separate them from free miRNAs. By subtracting the amount of miRNA present in the supernatant from the total input of miRNA, the miRNA loading efficiency was calculated. Then, the physical stability of vesicles and entrapped miRNAs in bilayer vesicles was measured by thermal stability method. Briefly, the thermal stability of entrapped miRNA into lipoplexes was evaluated during 48 hrs at various temperatures (4, 25, 37 and 42 °C) using gel (2 %) electrophoresis assay, as mentioned previously. The ability of liposome to retain the miRNA was determined as a function of rigidity and thermal stability of liposome versus temperature.

### Human mesenchymal stem cell culture

The hMSCs were grown in DMEM medium supplemented with 10 % fetal bovine serum (FBS), 100 units/mL penicillin, 100 µg/mL streptomycin and 2.5 µg/mL amphotericin B. The cells grew as adherent single layer. They were passaged at 80 %-90 % confluence. For all experiments the cells were used only at their exponential growth phase.

### hMSC treatments

The hMSC treatment groups included: empty liposomes, liposomal miRNAs (LP-miRNAs, lipoplexes), free miRNAs, and no treatment as control, according to the references (Pakunlu et al., 2006[14]; Wu et al., 2013[23]). 

### hMSC proliferation assay

Cellular proliferation under the influence of the studied formulations was assessed using the MTT assay. Briefly, the cells were seeded into 96-well plate and allowed settlement on the bottom of the plate for 24 hrs, and then were treated with an equal volume of fresh medium containing different concentrations of each formulation. The duration of incubation was 48 hrs. Then, 20 μL of MTT (5 mg/mL) was added into every well and incubated for 3 hrs, after which the supernatant was evacuated and 180 μL of DMSO was added to dissolve the crystals. Optical density values were recorded using Synergy TMHT multi-mode microplate reader (Biotek Instruments Inc, USA). 

### Transfection of hMSCs with LP-miRNAs 

hMSCs were seeded in 6-well plates (5×10^5^ cells/well) and cultured in DMEM medium supplemented with 10 % FBS overnight at 37 °C in a 5 % CO_2_ humidified incubator. The culture medium was then replaced with the medium containing no FBS. Each LP-miRNA was then added separately to each well at miRNA concentration of 100 nmol/L, according to the references. The cells were incubated at 37 °C for 4 hrs, and then transferred into 2 mL of fresh culture medium supplemented with 10 % FBS. All transfection experiments were performed in triplicate. FAM-miRNAs and LP-FAM-miRNAs were also used to evaluate cell uptake of FAM-LP-miRNAs and free FAM-miRNAS. The cells were then washed three times with PBS and fixed with paraformaldehyde solution. DAPI solution (0.125 μL/mL) was employed for 15 min for nuclear counterstaining. Cell transfection efficiency of miRNA was evaluated with a fluorescence microscope (Olympus, Japan) (Pakunlu et al., 2006[14]; Wu et al., 2013[23]; Ando et al., 2013[2]). 

### RNA isolation from the treated hMSCs 

Total cellular RNA was isolated from hMSCs grown to passage 3 and treated, using Ribo EX kit (Geneall, Daejeon, Korea). The quality and quantity of RNA content was evaluated using Nanodrop-2000 (Thermo Fisher Scientific, Waltham, MA, USA). Absorbance ratios and concentrations were determined as indicators of sample yield, quality and purity. The quality of total RNA was analyzed using gel electrophoresis. 

### Reverse transcription and cDNA synthesis

To evaluate primers by RT-PCR and to measure the expression of Parp1 by quantitative real-time PCR (QPCR), the isolated total RNA was transcribed into cDNA using the High Capacity cDNA Reverse Transcription Kit (RevertAid First Strand cDNA Synthesis Kit, Thermo Scientific, USA). Briefly, 1 µg of RNA was mixed with 1 µL of the random hexamer primer and added nuclease-free water (QIAGEN, Germany) up to 12 µL, according to the kit instructions. The tubes were then incubated in a thermocycler (Bio-Rad Laboratories, USA) at 65 °C for 5 min after which the tubes were placed on ice (4 °C) and the other reagents were added, as per the kit instructions. First strand cDNA was then amplified with the following program: 5 min at 25 °C, 60 min at 42 °C, and 5 min at 70 °C.

### QPCR

To determine the amount of mRNA levels of target gene (Parp1), QPCR was done using HOT FIREPol® Eva Green qPCR Mix (Solis Bio Dyne, Tartu, Estonia), according to the kit instructions and SYBR Green method. The relative expression of target genes was measured by Step One Plus Real-time PCR (Applied Biosystems, USA) in duplicate in a final volume of 20 μL using cycling parameters (3 min at 95 °C; 3 s at 95 °C; 20 s at 60 °C with the latter two steps repeated for 40 times), and was then quantified by the ΔΔ*CT* method. The expression of Parp1 mRNAs was normalized to GAPDH (Applied Biosystems; assay ID: Mm99999915_g1), which was the endogenous reference in the corresponding samples, and relative to the untreated control cells. The primer sequences used in QPCR were as follows: Parp1 primers F: “5'-CGAGTAGCTGATGGCATGG -3'”, R: “5' -GACGTCCCCAGTGCAGTAAT-3' (with product size 102 bp); and GAPDH primers F: “5'-GAGCCACATCGCTCTGACAC-3' ”, R: “5'-ATGTAGTTGAGGTCAATGAAGG-3'” (with product size 157 bp). 

### Statistical analysis

Each experiment was performed in three times and the data were presented as mean ± S.D, unless stated otherwise. Statistical analysis was performed using one way ANOVA and Student's t-test, if appropriate. In all cases, P values less than 5 % were regarded as indicative of significant difference.

## Results

### Characterization and determination of loading efficiency of lipoplexes (LP-miRNAs)

**Figure 2:**
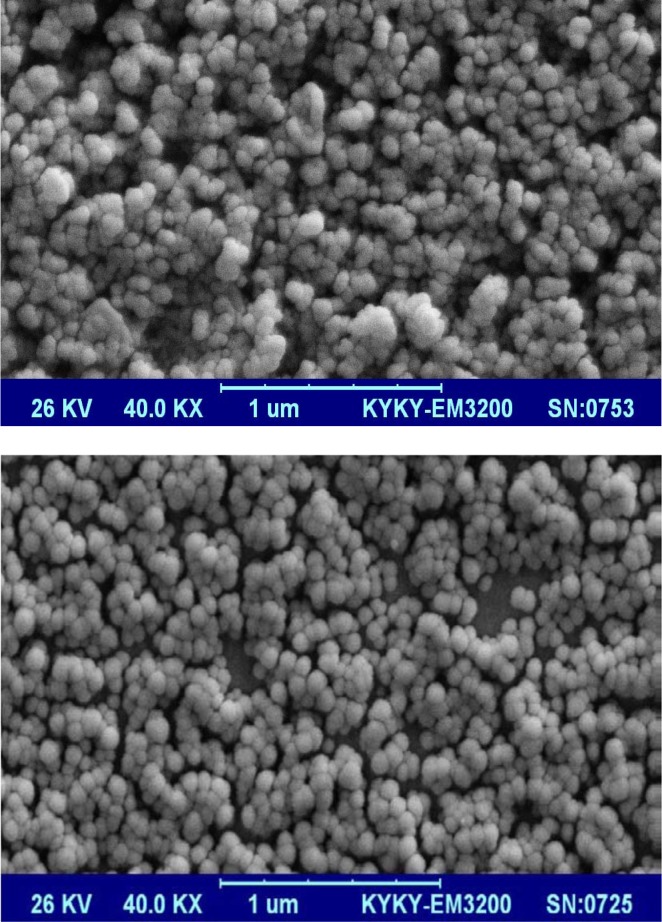
Scanning electron microscopic (SEM) image of lipoplexes. Top: liposome with miR-302a; Bottom: liposome with miR-34a. Magnification: ×10000

Before *in vitro* transfection experiments, we characterized the liposomes (Table 1(Tab. 1)), and then examined the SEM images of the lipoplexes (Figure 2(Fig. 2)). 

According to the results, incubation with miRNA generally led to decreased ζ-potential and increased size of vesicles. In all cases, PDI was less than 0.3 which implies minimal aggregations. miRNA incubation with liposomes increased liposome diameters for both formulations while it significantly (P value < 0.05) reduced the zeta potential up to 60 %. SEM images of lipoplexes (Figure 2(Fig. 2)) showed spherical shape with homogeneous size distribution without difference between the two lipoplexes. The size of LP-miRNA was estimated to be 100-140 nm. Lp-miRNAs exhibited better PDI value than blank liposomes, which indicates much lower tendency for agglomeration due to neutralization of the electrical charge of the particles. In assaying the interaction between miRNAs and liposomes in terms of loading efficiency, the migration of miRNA-loaded liposomes on gel electrophoresis was much lower than free miRNAs (Figure 1(Fig. 1), lanes 2-4). We found that the most suitable amount of miRNA encapsulated by liposomes is when the mass ratio of lipids/miRNA is 12.5. 

### Efficient cellular uptake of LPs 

**Figure 3:**
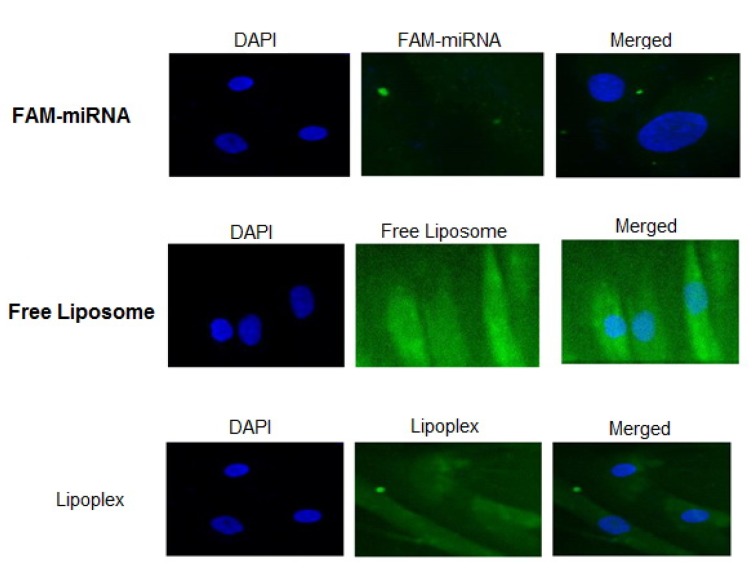
Fluorescent microscopic analysis of uptake of FAM-miRNA, free liposome, and lipoplexes 4 hrs after transfection. Cell nuclei were stained with DAPI (blue), and transfected cells were fixed with 4 % paraformaldehyde. Magnification: ×400

The miRNA loading ratio of the liposomes was around 80 %, and there was no difference in the ratio between miR-302a and miR-34a (Pakunlu et al., 2006[14]; Wu et al., 2013[23]). To confirm that the liposomes used in the present study can release their payloads inside the stem cells, we loaded liposomes with miRNAs labeled with fluorescent dye FAM. LP-miRNAs were prepared by mixing synthetic miRNA with empty liposome at a lipids/miRNA mass ratio of 12.5 (with 100 μg/ mg). Then, hMSCs were transfected with empty liposomes or free FAM-miRNA as controls and LPs containing FAM-miRNA at concentration of 100 nM (Wu et al., 2013[23]; Ando et al., 2013[2]; Endo-Takahashi et al., 2014[7]). Cellular uptake and localization was evaluated at 4, 24, and 48 hrs after transfection by fluorescent microscopy at 100× magnification (Figure 3(Fig. 3)). 

As observed, the LP-miRNAs had efficiently transfected the cells at 4 hrs of incubation. The transfection intensity in hMSCs with free miRNAs was found higher than that of LP-miRNAs; however, the prepared LPs can be used as an efficient delivery system *in vivo*. Moreover, microscopic examination displayed more accumulation of miRNAs in the cytoplasm of hMSCs even though miRNAs were observed in the nucleus, too. It was previously observed that MPEGylated liposomes fluently penetrate into the cytoplasm and nuclei of proliferating cell, making them an attractive vehicle for both cytoplasmic and nuclear delivery of miRNAs (Pakunlu et al., 2006[14]; Endo-Takahashi et al., 2014[7])*.*

### Are liposomes cytotoxic for human mesenchymal stem cells? 

**Figure 4:**
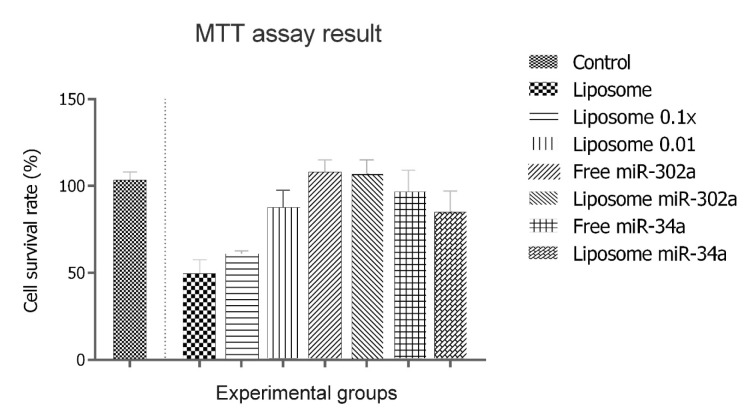
Evaluation of cell viability percentage for blank liposome prepared, free miRNA, and lipoplexes

Initially, hMSCs were treated with concentrations of empty liposome (1-0.01 mg/ mL total lipid, for 72 hrs) (Pakunlu et al., 2006[14]; Wu et al., 2013[23]) and their survival was measured by MTT assay in comparison to untreated control cells (Figure 4(Fig. 4)).

The results showed that liposome-incubated hMSCs exhibit significantly reduced level of viability (~50 %, p value < 0.05), when compared to untreated control. However, LP-miRNA treatment brought up the survival to the control level. Interestingly, the cells transfected with free miR-302a or LP-miR-302a showed a level of growth rate about or even higher than the control (about 115-110 % of control, p value < 0.05). It implies that LP-miR-302a and free miR-302a transfections would preserve the proliferation capacity of hMSCs and even more could naturalize the toxic effect of empty liposome. MiR-34a treatments could naturalize the toxic effect of empty liposome and significantly increase viability of hMSCs (p value < 0.05), but significantly less than miR-302a groups (p value < 0.05).

### Increased expression of Parp1 in miR-302a treatments

**Figure 5:**
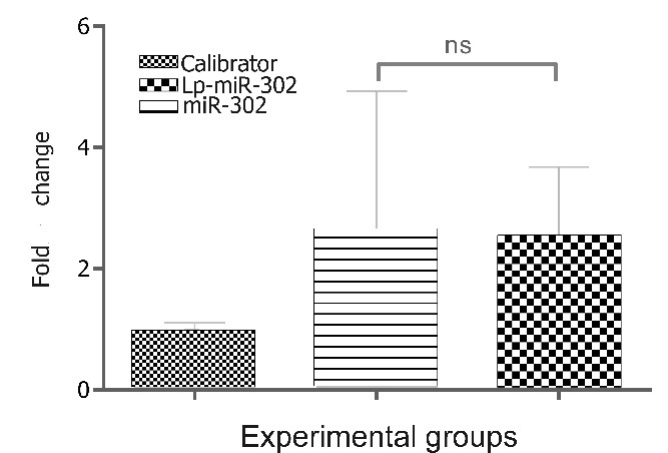
The relative expression of parp1 after miR-302a transfection with and without liposome. The Parp1 levels were transformed into quantities using the formula 2^-ΔΔCt^. Despite increased level of Parp1 expression, no significant (ns) difference was observed between free miR-302 and LP-miR-302. Error bars represent standard deviation.

Epigenetic modification by Parp1 is necessary and required at early stage of somatic cell reprogramming. Parp1 acts to maintain or induce an active epigenetic state by key pluripotency factors. Past studies showed that Parp1 acts as core component of the pluripotency network and as determinant of the developmental plasticity of stem cells. Accordingly, Parp1 plays a critical role in safeguarding stemness state (Doege et al., 2012[6]; Roper et al., 2014[19]). To find the mechanism of interaction between ectopic expression of miR-302a and epigenetic factor Parp1 in hMSCs, the fold change of Parp1 expression was measured, which was increased considerably (a fold change of ~ 2.58) in both groups when compared to the untreated control group (Figure 5(Fig. 5)).

It had been found that through the interaction with Parp1 pathway, miR-302a expression potently blocks human pluripotent stem cell (hPSC) differentiation and facilitates their self-renewal (Doege et al., 2012[6]; Roper et al., 2014[19]; Jiang et al., 2015[11]).

### Decreased expression of Parp1 due to miR-34a treatments

**Figure 6:**
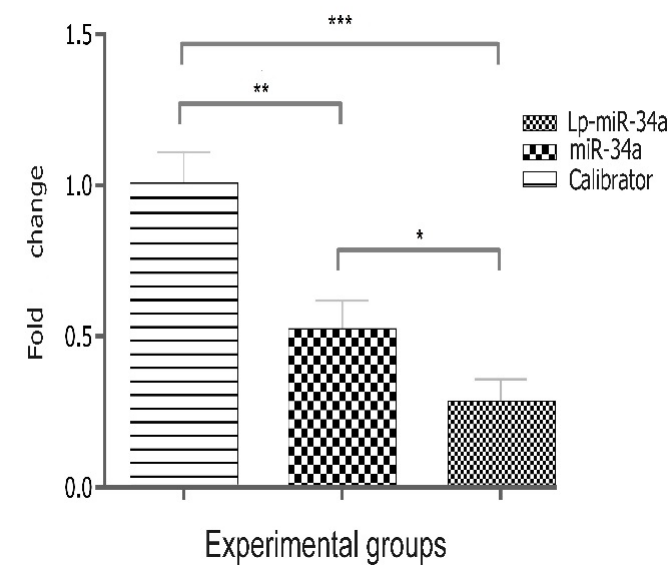
The relative expression of Parp1 after miR-34a transfection with and without liposome. The Parp1 levels were transformed into quantities using the formula 2^-ΔΔCt^. Error bars represent standard deviation. *P value < 0.05; **P value < 0.0017; ***P value < 0.0002.

Previously, it was reported that miR-34a induces differentiation in pluripotent stem cells which is accompanied by reduced self-renewal and proliferation. It was also reported that miR-34a induces senescence in progenitor stem cells through the interaction with Sirt1, the closest partner of Parp1 acting as epigenetic switches (Yu et al., 2015[24]; Zhao et al., 2010[25]; De Bonis et al., 2014[5]). Herein, the expression of Parp1 in hMSCs transfected with LP-miR-34a and free miR-34a at miRNA concentration of 100 nM was significantly reduced (the fold change ~ 0.5-0.25, in comparison with the control, p < 0.0017 (Figure 6(Fig. 6)). 

**Figure 7:**
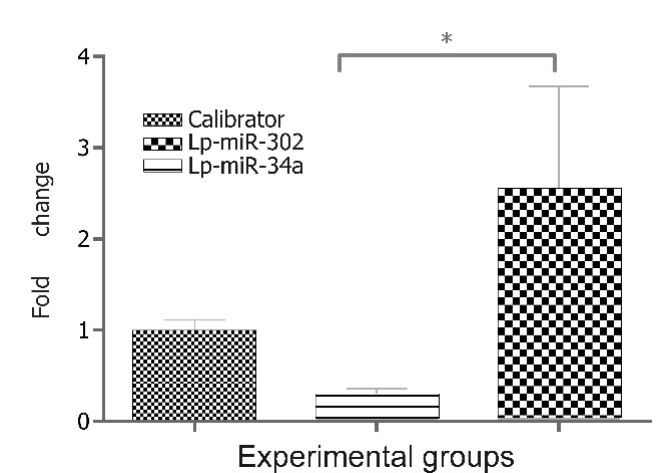
Comparison of relative fold change of Parp1 between miR-34a and miR-302a with similar liposome formulations. The miR-34a lipoplex significantly reduced Parp1 expression. Error bars represent standard deviation of the mean (SD). * P < 0.011.

It is probable that miR-34a interacts with Parp1 through the Sirt1 pathway whereby would potently block self-renewal of hMSCs and facilitate differentiation (Son et al., 2013[20]; Lee et al., 2013[12]; De Bonis et al., 2014[5])*.* Comparing miR-302a with miR-34a (Figure 7(Fig. 7)) demonstrated that the former is significantly different from the latter in terms of fold change in Parp1 expression (P < 0.011). 

## Discussion

Mesenchymal stem cell senescence clearly defines stem cell exhaustion as an underlying mechanism of body aging and reduced potential of hMSCs in tissue regeneration. The hypothesis declares a process whereby various stressors accumulate, finally inducing permanent arrest of cell proliferation (Pourrajab et al., 2013[15], 2014[18]; Wagner et al., 2008[21])*.* *In vitro* expansion of MSCs is limited so that after a certain number of cell divisions, they enter senescence which can be characterized by reduced potency of proliferation and somehow by reduced expression of pluripotency determinant factor Parp1 (Wagner et al., 2008[21]; Gang et al., 2007[9]; Doege et al., 2012[6]; Roper et al., 2014[19]). This phenomenon is described in murine and human MSCs which exhibit reduced self-renewal and differentiation potential upon prolonged *in vitro* culture. There is an ineluctable epigenetic clock in these cells which up-regulates cell-cycle inhibitor pathways and collectively generates a senescent differentiated progeny that rapidly acquires cell cycle arrest and the senescent phenotype. As a promising tool for cell therapy and regenerative medicine, we need to culture and expand hMSCs prior to their application (Pourrajab et al., 2014[18]; Wagner et al., 2008[21]). Additionally, reprogramming-induced senescence (RIS) is a barrier of reprogramming, triggered by up-regulation of cell-cycle inhibitor pathways. Senescence impairs successful reprogramming of somatic cells into iPSCs, which renders reprogramming slow and stochastic (Son et al., 2013[20]; De Bonis et al., 2014[5]). De-condensation of whole chromatin is one of the mechanisms behind RIS wherein enforced expression of four reprogramming factors OSKM (Oct4, Sox2, Klf4, and c-Myc) is closely associated with the activation of INK4A/ARF pathway, which causes cellular senescence or apoptosis, finally lowering the reprogramming efficiency (Doege et al., 2012[6]; Lee et al., 2013[12]; Zhao et al., 2010[25])*.* There is supposed early role of Parp1 in stem cell pluripotency-rejuvenation process whereby epigenetic function of Parp1 in the reprogramming process supplements pluripotency control. Parp1 function is required for the heterogeneous expression of critical pluripotency factors OSKM and reversible epigenetic regulation of repressed chromatin domains, and affects chromatin accessibility for reprogramming factors. Parp1 protects them from progressive epigenetic repression by occupying key pluripotency genes. In the absence of Parp1, pluripotent stem cells would exhibit a decrease in ground state pluripotency as they cannot maintain the typical proliferation capacity characteristic of the self-renewal state (Doege et al., 2012[6]; Roper et al., 2014[19]; Endo-Takahashi et al., 2014**[7]**)**. **Furthermore, cell fate decisions and the development of cellular phenotypes are controlled by a regulatory system of small (≈22 nt) non-coding microRNAs (miRNAs), which usually inhibit but may rarely increase gene expression at the pre- or post-transcriptional levels (Pourrajab et al., 2014[17], 2015[16]). Previously, it was reported that a total of 28 miRNAs can prevent stress-induced growth arrest and senescence in human stem cells. This group primarily includes stem cell-specific miRNAs miR-17-92 cluster, as well as miR-302 cluster. It was found that over-expression of these miRNAs rescues human primary cells from Ras-induced senescence, achieved by prevention of stress-induced up-regulation of senescence pathway (Borgdorff et al., 2010[4]; Fareh et al., 2012[8]). Researchers also revealed that the miR 302-367 cluster drastically affects self-renewal and proliferation properties of tumor cells through molecular pathways and consequent disruption of the Nanog network repression. That study implies that the miR-302-367 cluster exhibits tumor suppressor function in malignancies, and is strongly induced during serum-mediated stemness suppression. Stable miR-302-367 cluster expression is sufficient to preserve the stemness signature, self-renewal, and cell infiltration within a normal primary stem cell but not to suppress burst of proliferation in tumor tissues (Fareh et al., 2012[8]; Aranha et al., 2010[3]). Theoretically, a stem cell-specific miRNA such as miR-302 can target genes involved in differentiation or senescence as part of a shared pathway. Noteworthy, the repression by some miRNAs is especially strong when the level of the intracellular target mRNA is low, but weak when the level is high, meaning that a miRNA can act as a switcher or a fine-tuner depending on the target mRNA level (Lee et al., 2013[12]; Gao et al., 2015[10]). Moreover, different mRNAs that share the same target site for a specific miRNA can compete for binding to the miRNA and thereby mutually influence the expression of each other. This may result in different outcomes of the same miRNA in distinct cell types due to expression of a diverse set of mRNA targets (Pourrajab et al., 2014[17], 2015[16]). Accordingly, senescence of hMSC might be addressed to reduced expression of stem cell-specific miR-302 cluster (e.g. miR-302a). These findings place miR-302a at the genetic-epigenetic interface of pluripotency networks, fine-tuning the transcriptional heterogeneity and thereby determining the developmental plasticity of human stem cells (Lee et al., 2013[12]; Gao et al., 2015[10]). Supportively, our results showed that ectopic expression of miR-302a induces Parp1 expression which would accelerate pluripotency gene modifications into an open chromatin configuration.

Previously, it was shown that miR-302 ectopic expression can attenuate reprogramming-induced senescence, protecting expression of reprogramming factors. These raise the possibility that ectopic expression of miR-302a may participate in the regulation of epigenetic machinery in a manner that favors reprogramming, at least in part through the expression of Parp1 because both miR-302a and Parp1 protect pluripotency genes during reprogramming (Doege et al., 2012[6]; Son et al., 2013[20]; De Bonis et al., 2014[5]). Furthermore, senescence of MSC might limit their therapeutic applications and thus, analysis of MSC in vitro senescence is crucial for basic research as well as for application of new strategies in cellular therapy (Pourrajab et al., 2013[15], 2014[18]; Wagner et al., 2008[21])*.* Importantly, particular miRNAs (e.g, miR-302a or miR-34a), may serve as tumor suppressors by regulating processes essential for cancer development or through simultaneous regulation of multiple biological pathways, including proliferation, survival, apoptosis, and metastasis. Herein, miRNA mimics may represent a powerful therapeutic strategy in cell therapy of cancers or regenerative medicine. Over the past decade, most researches involving ectopic expression of miR-34 have focused on its role in inducing apoptosis, cell-cycle arrest or senescence. However, studies analyzing miRNA-34a function in hMSC pluripotency and self-renewal are limited and inconsistent. It has been recently reported that suppression of somatic cell reprogramming into pluripotent cells is due to the repression of pluripotency genes by miR-34a which induces stem cell differentiation (Yu et al., 2015[24]; Zhao et al., 2010[25]; De Bonis et al., 2014[5]). Noteworthy, Sirt1 and Parp1 both are repressors of p53 whose transcriptional function induces miR-34a expression. Intriguingly, miR-34a acts as a senescence switcher by directly targeting Sirt1, the key of longevity pathway Sirt1/PCG-a/Foxo/ IGF-1. It was previously supposed that ectopic expression of miR-34a may switch off the Parp1/Sirt1 system, leading to p53 over-activation and aggravation of senescence pathways (Pourrajab et al., 2015[16]). 

Here, we attempted to understand whether ectopic miR-302a and its counterpart miR-34a affect hMSC pluripotency and proliferation. Transfection protocols were used to assay the contribution of ectopic miRNAs to survival and expression of genes catalyzing epigenetic modification necessary for pluripotency markers. In our formulation, DSPE-MPEG and DOTAP are cationic lipids efficiently used in the past for gene therapy (Pakunlu et al., 2006[14]; Wu et al., 2013[23]; Ando et al., 2013[2]; Endo-Takahashi et al., 2014[7]). DOTAP has shown high transfection activities *in vitro*, since it confers a positive surface charge to the LPs which in turn facilitates the interaction between LPs and the negatively-charged cell membrane. Accordingly, cholesterol, as a neutral lipid, gives stability to the LP structure and improves transfection efficiency *in vitro *and* in vivo*, facilitating cellular uptake (Wu et al., 2013[23]). DSPE-MPEG was used to increase the stability and circulation time of LPs *in vivo* (Pakunlu et al., 2006[14]). *In vitro* cell transfection was assessed by fluorescent microscopy images which showed that both free miRNAs and LP-miRNA were able to transfect hMSCs efficiently. Transfer of miRNAs was observed even in the nuclei of hMSCs, which is important because endogenous miRNAs have been detected in the cell nucleus (Pakunlu et al., 2006[14]; Wu et al., 2013[23]). Parp1 expression was found to be profoundly affected by ectopic miR-302a and miR-34a. Expressed miRNAs indirectly target the genes of pluripotency and self-renewal by modulating epigenetic determinant Parp1. The determinant is usually silenced or down-regulated in many senescent or differentiated cells (Doege et al., 2012[6]; Roper et al., 2014[19]; Son et al., 2013[20]). We showed that ectopic expression of miR-302a (a potent inhibitor of stem cell differentiation) can preserve hMSC proliferation potency even in the presence of a stressor, i.e., liposome. However, miR-34a ectopic expression exhibited an opposite effect which could not preserve cell proliferation in the presence of a stressor or even bring growth rate up to the normal level. Herein, data provide evidence that the positive effects of miR-302 and miR-34a on pluripotency control are molecularly associated with the repression of Parp1 which in turn is associated with the expression of Sirt1, p53, p21, and p16 pathways (Pourrajab et al., 2014[17], 2015[16]; Yu et al., 2015[24]). Our findings suggest that ectopic miR-302a and miR-34a may be used for maintaining pluripotency. The distinct effects of miR-302a and miR-34a on pluripotency may be not only dependent on Parp1 but also on its close partner Sirt1. The cellular advantage of ectopic miR-34a in repressing Parp1 can be addressed to its ability to enhance resistance to cellular stress (Agostini and Knight, 2014[1]; Yu et al., 2015[24]). 

In summary, our hypothesis is that miRNA-mediated therapy may mediate pluripotency regulation and add an additional layer of control over network regulation or providing a fine-tuning switch or mechanism to enforce the cell-fate decisions in response to external stimuli. In this regard, the miR-302-367 cluster expressed at high level in hPSCs has been emerged as a desirable participant in maintenance of stemness, cell proliferation and cell cycle which among others causes a delay in early human embryonic stem cell differentiation. It is known that miR-34 up-regulates p53 to down-regulate a set of genes involved in cell cycling of tumor cells. Parp1 was up-regulated in transfected hMSCs and supposed to play a key role in erasing somatic epigenetic signatures, as typified by DNA methylation or histone modification (Pourrajab et al., 2014[17], 2015[16]). These results therefore expand the regulatory role of miRNAs and provide a pharmacologic rationale for the use of miRNAs as an efficient therapeutic approach. Generally, the present study was designed to determine the effect of ectopic miR-302a and miR-34a on preservation of hMSC pluripotency. Given the challenges posed by the use of mesenchymal stem cells in regenerative medicine, the results showed that the roles of miR-302a and miR-34a in preserving pluripotency of hPSCs can be in part through Parp1 pathway. These findings may contribute to the development of defined and scalable systems containing ectopic miRNA components which are fundamental and prerequisite for production of desirable cell types including hMSC and hiPSC.

## Conflicts of interest

The authors declare that they have no conflict of interest.

## Acknowledgement

The authors are grateful to Cardiovascular Research Center and also Central Laboratory of International Campus of Shahid Sadoughi University of Medical Science in Yazd, Iran. Also we thank Ms Fatemeh Montazeri, a researcher in Research and Clinical Center for Infertility, Shahid Sadoughi University of Medical Science in Yazd, Iran, for her technical support. This work was supported by Shahid Sadoughi University of Medical Sciences, Yazd, Iran as part of an MSc thesis project.

## Figures and Tables

**Table 1:**
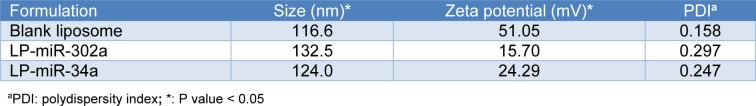
Characterization of the prepared liposomes
